# Legislating Fear: How Immigration Status Mandates Threaten Public Health

**DOI:** 10.5811/westjem.42065

**Published:** 2025-03-24

**Authors:** Peter Sangeyup Yun, Lindsey Williams, Janice Blanchard

**Affiliations:** George Washington University, Department of Emergency Medicine, Washington, District of Columbia

A mother hesitates in a Texas emergency department, cradling her feverish child, unsure whether to proceed with treatment. Her fear isn’t just about the illness—it’s about the question she knows the hospital will ask: “What is your citizenship status?”

With the implementation of Executive Order GA-46 in Texas and Florida’s Senate Bill 1718 earlier this year, such fears have become widespread. These laws mandate that hospitals gather immigration status information from patients upon admission or registration. Proponents claim this ensures accountability for public resources, but the truth is far more complex and potentially harmful.

Contrary to the assumptions underpinning these policies, undocumented immigrants use healthcare services far less frequently than US citizens or other migrant groups. Research by Pourat et al revealed that California’s undocumented population accessed significantly fewer health services compared to US-born residents.^1^ Another study demonstrated that immigrants, whether documented or not, incur lower per capita healthcare expenditures than their US-born counterparts. Annual expenditures were $1,629 per undocumented immigrant, $3,795 per documented immigrants, and $6,088 per US-born individual.^2^ These findings counter the myth that immigrants disproportionately burden the healthcare system. Furthermore, these policies burden the healthcare system by increasing costs to hospitals. According to Florida Bill 1718, hospitals will be required to collect data, provide analysis, and generate both quarterly and annual reports. This data collection and analysis require both human and financial resources. For institutions operating at low margins such as rural hospitals or safety-net hospitals, these policies place undue strain and provide questionable benefit.^3^

The harm caused by these laws at all levels is undeniable. Health and social disparities already disproportionately affect undocumented families, who face higher barriers to care. Studies show that these populations experience worse physical and mental health outcomes compared to the general US population.^4^ Laws targeting immigrant communities exacerbate these disparities, creating an environment of fear that discourages them from seeking care, even in life-threatening emergencies.^5^

Supporters of these policies argue that patients are not required to answer questions about their immigration status and that responses remain confidential under HIPAA. However, this reassurance often fails to reach the patient. Patients may not understand their rights, and the very act of asking these questions can sow mistrust. Delayed or forgone care due to fear can lead to worsened health outcomes, driving up costs for more severe and complex treatments.^6^

These laws impact not only undocumented adults but entire families. In Texas, more than one million US-born children live in households with at least one undocumented family member. When parents feel discouraged from seeking medical care, it can place their children’s health at risk as well.^7^ Such policies disproportionately affect our most vulnerable populations, posing challenges to individual lives and the well-being of entire communities.

Hospitals, as trusted institutions, are deeply affected by these challenges. Trust forms the backbone of public health, and its erosion can have widespread consequences for communities. Access to care should not be limited by immigration status. As physicians, our ethical responsibilities guide us to protect and advocate for all patients, regardless of their legal status.^8^ Our mission rises above political differences, grounded in the shared need for equitable and compassionate care.

To mitigate the harm caused by these laws, we propose the following immediate actions:

1. **Standardized Messaging:** Develop hospital-wide policies and training for staff to ask citizenship questions in a non-threatening, scripted manner. For instance: “You are not required to answer, and this is a question we ask everyone: Are you a citizen of the United States?” National physician organizations, such as the American Medical Association, should issue clear guidelines for implementing these laws ethically.
2. **Interpreter Training:** Train interpreters to deliver sensitive information in a culturally appropriate and non-judgmental way, ensuring equitable communication for patients with limited English proficiency.
3. **Community Outreach:** Collaborate with local organizations to educate communities about their rights, emphasizing that patients are entitled to emergency care regardless of immigration status.
4. **Patient Education:** Provide multilingual informational sheets or badges explaining the new laws and patients’ rights and display them prominently in hospitals and clinics.
5. **Community Partnerships:** Work with advocacy groups and pro bono organizations to disseminate accurate information and provide resources for undocumented patients.
6. **Data Transparency:** Advocate for access to the data collected under these mandates. If this data is to be gathered, it should serve public health goals, enabling physicians and policymakers to better understand trends and improve care for underserved populations.

These measures are an important step forward, but much more needs to be done. A lasting solution requires dismantling policies that create barriers to healthcare for vulnerable communities. As medical professionals, we are guided by the principles of beneficence, nonmaleficence, and justice. To truly honor these values, we may need to extend our advocacy beyond the clinic, emergency department, or operating room and engage with our communities. Our voices carry weight, and we must continue to affirm that immigration status should never stand in the way of accessing care.
